# Multiple sequential antibody-associated syndromes with a recurrent mutated neuroblastoma

**DOI:** 10.1212/WNL.0000000000002945

**Published:** 2016-08-09

**Authors:** Ali Amini, Bethan Lang, Dominic Heaney, Sarosh R. Irani

**Affiliations:** From the Nuffield Department of Clinical Neurosciences (A.A., B.L., S.R.I.), University of Oxford; and National Hospital for Neurology and Neurosurgery (D.H.), London, UK.

A 5-year-old girl was diagnosed with opsoclonus-myoclonus syndrome (OMS) and a para-aortic neuroblastoma (NB) (figure 1A). Urinary catecholamines were elevated; NB biopsy confirmed no *MYCN* gene amplification. Serum showed Hu antibodies without hippocampal neuron surface antibodies (NSAbs) (figure 1B) and, of note, serum inhibited proliferation of cultured NB cells (figure 1C). After partial resection and chemotherapy, she progressed well in mainstream education.

**Figure:**
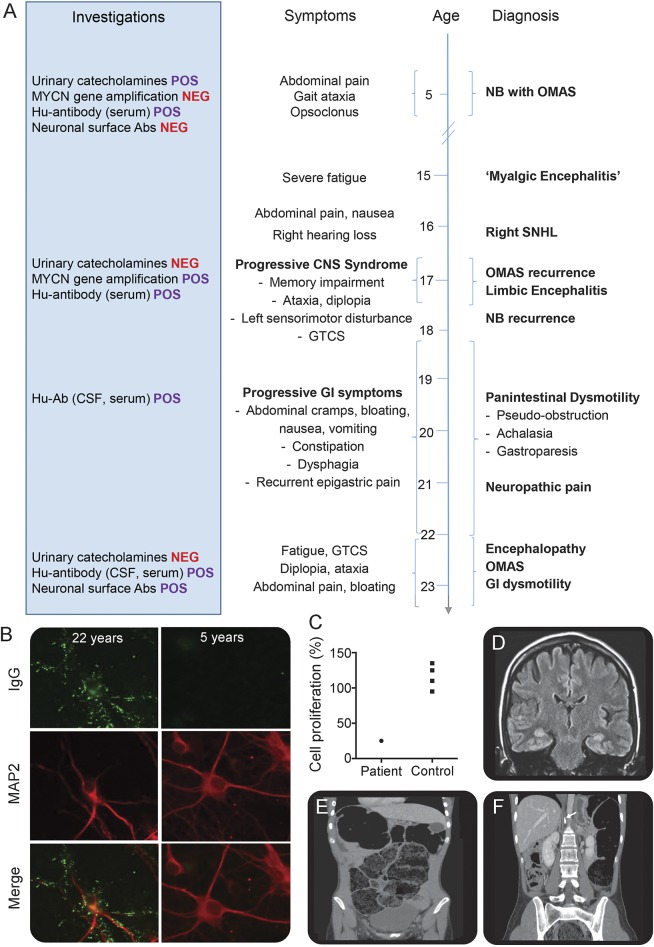
Clinical, serologic, and radiologic correlations in relapsing opsoclonus-myoclonus (A) Chronology of symptoms, diagnoses, investigation results, and treatment including clinical response. (B) Immunolabeling of MAP2-positive hippocampal neurons in culture with our patient's plasma. IgG antibodies (green) from her sera aged 22 years (top panels) produced widespread labeling of the surface of hippocampal neurons and dendrites (identified by MAP2 staining, red), not identified in her plasma from 5 years of age (bottom panels). (C) Cultured NB cell proliferation is relatively inhibited by our patient serum. (D) Coronal view fluid-attenuation inversion recovery brain MRI with bilateral medial temporal lobe and mild right precentral gyrus high signal. (E) Coronal CT sections show chronic constipation and pseudo-obstruction from intestinal dysmotility. (F) Coronal CT demonstrating residual aortocaval mass (arrow) with calcification typical of an NB. GI = gastrointestinal; GTCS = generalized tonic-clonic seizure; Hu-Ab = Hu antibody; IgG = immunoglobin G; MAP2 = microtubule-associated protein 2; NB = neuroblastoma; NEG = negative; OMAS = opsoclonus-myoclonus ataxia syndrome; POS = positive; SNHL = sensorineural hearing loss.

A decade later, she developed weight loss and abdominal pain followed by sudden right-sided sensorineural hearing loss with a gradually progressive syndrome of ataxia and opsoclonus, which responded transiently to corticosteroids. Subsequently, a generalized seizure, ataxia, multidirectional diplopia, and left arm sensorimotor disturbance prompted evaluation. This revealed bilateral medial temporal and mild right precentral gyrus high signal on MRI (figure 1D), moderately diffuse slowing on EEG, and serum Hu antibodies. Serum antibodies to GAD, Yo, or Ri, the VGKC complex, the NMDA, GABA_A_, GABA_B_, glycine α1, and AMPA receptors, tumor markers, and urinary catecholamines were not detected. Abdominal magnetic resonance and metaiodobenzylguanidine scintigraphy suggested NB recurrence. On this occasion, biopsy showed *MYCN* gene amplification and chromosome 1p deletion.

IV methylprednisolone (IVMP) plus NB debulking and radiotherapy allowed her to return to college feeling the best she had in 3 years, with resolution of signs and with normal neuroimaging.

One year later (figure 1A), she developed vomiting, dysphagia, and abdominal bloating. Serial imaging confirmed esophageal, colonic, and small bowel dilation (figure 1E) with stable residual tumor appearances (figure 1F). This paraneoplastic chronic intestinal pseudo-obstruction (CIPO) remained refractory to medical therapies. Hu antibodies were found in serum and CSF and were the only reactivity observed on myenteric plexus staining. Alpha-3 acetylcholine ganglionic receptor antibodies were absent.

At age 22, worsening panintestinal dysmotility, cognition, and recurrent opsoclonus culminated in another seizure. Investigations showed new frontotemporal slow-wave activity, restricted CSF oligoclonal bands, and now, serum hippocampal NSAbs (figure 1B). IV methylprednisolone reduced frontotemporal activity and CSF Hu antibody titers, with transient symptom amelioration. Plasmapheresis achieved more sustained gastrointestinal and neurologic remission, permitting university studies continuation.

## Discussion.

This case raises several important clinical and molecular observations with intriguing implications for mechanisms of autoantibody generation.

First, there is the striking co-occurrence of 4 autoimmune syndromes associated with Hu antibodies and NB. These are likely to be autoantibody-mediated and 3 are often paraneoplastic. Adult paraneoplastic OMS and limbic encephalitis (LE) with Hu antibodies are associated with breast cancer and small cell lung cancer. Usually, they show limited responses to immunotherapy.^1,2^ By contrast, pediatric OMS typically associates with an NB of favorable prognosis and cytogenetics (absence of *MYCN* amplification) but rarely the Hu antibodies detected in our patient.^3,4^ Pediatric Hu antibody–associated LE is usually nonparaneoplastic,^4^ whereas paraneoplastic Hu antibody–associated CIPO is usually reported with small cell lung cancer, with only rare reports in childhood NB. Paraneoplastic CIPO is important to differentiate from oncologic iatrogenic complications given its potential response to immunotherapy.^5^ Abrupt-onset sensorineural hearing loss is usually an autoimmune steroid-responsive disorder, and has also been associated with Hu antibodies.^6^ OMS itself has only rarely been associated with NSAbs.^7^

Second, cytogenetic and biochemical characteristics of our patient's NB altered on recurrence. An alteration in the tumor biology, such as the de novo observed *MYCN* amplification and 1p deletion, may have broken immunologic tolerance and generated antibodies against NB-expressed antigens, also expressed in the hippocampi (causing LE) and the myenteric plexus (producing CIPO). This alteration may also be linked to the cessation of catecholamine secretion. Furthermore, a chromosome 1p deletion in childhood NB would usually signify poor prognosis, but since its partial resection, our patient has already survived 7 years and completed higher education. Indeed, our patient's serum inhibited proliferation of NB cells in culture. Collectively, these findings strengthen the established concept that an autoimmune response against the tumor may suppress its growth, as hypothesized in Lambert-Eaton myasthenic syndrome and classic anti-Hu antibody syndromes.^2^ Also, maybe this ongoing antitumor response can expose neoantigens and lead to epitope spread. Ideally, this should be examined longitudinally using several serum and CSF samples but, unfortunately, these were not available from our patient.

Third, to our knowledge, this is the longest lag to the second presentation of NB with OMS and urges ongoing tumor vigilance during patient follow-up. Delayed recurrence of OMS after 9 years, and Hu antibody–associated LE 6 years after pediatric NB with OMS have been reported, with incomplete cytogenetic data.^3^

Our case should prompt research correlating tumor cytogenetics and clinical progression in recurrent or spontaneously remitting tumors as a method to elucidate mechanisms linking antitumor and nervous tissue autoimmunity, tolerance mechanisms, and in ultimately identifying predictive signatures to guide therapy.

## Supplementary Material

Accompanying Editorial
