# Supplementary material for: Multiple sequential antibody-associated syndromes with a recurrent mutated neuroblastoma
Source: Neurology. 2016 Aug 9;87(6):634–6. doi: 10.1212/WNL.0000000000002945 (PMC4977371; doi:10.1212/WNL.0000000000002945)
Supplement: Accompanying Editorial [file supp_87_6_634__index.html]

Accompanying Editorial 

# Multiple sequential antibody-associated syndromes with a recurrent mutated neuroblastoma

## Accompanying Editorial

**Neurology® data supplements are not copyedited before publication. Published editorials and translations have been copyedited.  
 © 2016 American Academy of Neurology.  
  
 Files in this Data Supplement:**

- Accompanying Editorial - PDF
