# Supplementary material for: Multiple sequential antibody-associated syndromes with a recurrent mutated neuroblastoma
Source: Neurology. 2016 Aug 9;87(6):634–6. doi: 10.1212/WNL.0000000000002945 (PMC4977371; doi:10.1212/WNL.0000000000002945)
Supplement: Accompanying Editorial [file supp_WNL.0000000000002945_560.pdf]

The origins and progression of CNS autoimmunity

Nature, nurture, and tumor

Russell C. Dale, MRCP, PhD
Ming Lim, MD, PhD

Correspondence to Dr. Dale: russell.dale@health.nsw.gov.au

Neurology® 2016;87:560–561

Neuroblastoma is the most common extracranial solid cancer of childhood, accounting for 15% of cancer deaths in children.[1] A small proportion (up to 4%) of children with neuroblastoma develop opsoclonus-myoclonus ataxia syndrome (OMAS), a paraneoplastic autoimmune CNS disorder.[2] Of note, neuroblastoma associated with OMAS is frequently of low stage, incurring a better prognosis,[3] suggesting that this tumor-triggered autoimmunity may also have a protective role.

In this issue of *Neurology*®, Amini et al.[4] describe an intriguing and complex case that provides further glimpses at this important facet of CNS autoimmunity. The case in question initially presented at age 5 with OMAS, a para-aortic neuroblastoma, and anti-Hu antibodies; the tumor was successfully treated.

Ten years later, the patient presented with a complex autoimmune CNS syndrome, with limbic encephalitis features plus opsoclonus and sensorineural deafness. The tumor had recurred and mutated, now with *MYCN* gene amplification (having previously been negative) and chromosome 1p deletion. Further tumor therapy plus IV methylprednisolone induced a surprisingly good recovery from the neurologic illness, allowing return to full time academic education. The patient developed a further suspected autoimmune complication 1 year later (chronic intestinal pseudo-obstruction), and then had a recurrence of the CNS syndrome, which responded to corticosteroids and plasma exchange, resulting in a return to normal functioning.

In this patient, the breadth of evolving, overlapping, and relapsing nervous system features of suspected autoimmune origin, OMAS, limbic encephalitis, reversible sensorineural deafness, and pandysmotility syndrome (brain, ear, autonomic gut involvement) is highly unusual, although the co-occurrence of autoimmune nervous system syndromes is increasingly recognized, such as myasthenia gravis plus neuromyelitis optica and anti-NMDA receptor encephalitis plus CNS demyelination syndromes.[5,6]

The most interesting aspect of this case is the mutating neuroblastoma, which alongside other potential, yet unidentified, host factors is likely to have generated the diversity of autoimmune syndromes in this patient. Neural crest tumors make intellectually appealing triggers of paraneoplastic CNS disease, given the "neuronal-like" features of the tumor. Although there is currently little evidence of a "cell surface antibody" in the majority of patients with OMAS,[7] the consistent immunotherapy responsiveness and histopathologic evidence of inflammatory infiltrate observed in neuroblastoma tumors associated with OMAS strongly support an autoimmune response both toward the tumor and also against the brain.[8] Furthermore, this immunotherapeutic tumor effect has been successfully used in treating neuroblastoma with antibodies or, more recently, engineered T cells against highly expressed tumor antigen such as disialoganglioside (GD2).[1,9]

The impressive reversal of symptoms with only modest immune suppression at each relapse in this case is more typical of that seen in cell surface antibody–associated syndromes rather than classic paraneoplastic syndromes associated with onconeuronal antibodies (such as anti-Hu antibodies). It is conceivable, even likely, that this patient had a reversible immunologic process (humoral or cellular) throughout the disease course that was not detectable by current standard measures or antibody assays.

The tumor mutation at the time of relapse in the patient reported by Amini et al.[4] suggests that alteration of the cellular nature of the tumor is likely to have "reactivated" the paraneoplastic autoimmune CNS syndrome, by means of exposure of the host to "cryptic antigens" causing resultant loss of prior established immune tolerance, as hypothesized by the authors. This observation augments our understanding of the origins of autoimmune nervous system disease in a similar way to the recent recognition that the destructive pathology of herpes simplex virus encephalitis can provoke a secondary autoimmune response associated with anti-NMDA receptor antibodies or other cell surface antibodies,[10] with the putative unifying process being the "release of previously cryptic neuronal antigens in an inflammatory milieu."

See page 634

From the Institute for Neuroscience and Muscle Research (R.C.D.), the Children's Hospital at Westmead, University of Sydney, Australia; and Children's Neurosciences (M.L.), Evelina London Children's Hospital at Guy's and St Thomas' NHS Foundation Trust, King's Health Partners Academic Health Science Centre, London, UK.

Go to Neurology.org for full disclosures. Funding information and disclosures deemed relevant by the authors, if any, are provided at the end of the editorial.

© 2016 American Academy of Neurology

© 2016 American Academy of Neurology. Unauthorized reproduction of this article is prohibited.

The careful longitudinal serum antibody analysis at presentation and through the relapsing phases of the disease in this case provides further insights into the natural history and evolution of the autoimmune process. Only anti-Hu antibodies were present throughout the clinical course, which are unusual in pediatric OMAS, and only rarely reported in children with inflammatory brain disease.[11] Testing for a panel of cell surface antibodies was negative, and initial screening for a cell surface antibody using cultured live hippocampal neurons was negative at disease onset, although became positive in the relapsing stages of the illness over a decade after presentation. The fact that the patient only developed a potentially pathogenic cell surface antibody during the relapsing phase of disease further emphasizes the evolving nature of disease in this patient, hypothetically due to "epitope spreading" of the autoimmune response, potentially triggered by the mutating tumor. What is also intriguing is that during the early stages of disease, despite having no immunoglobulin binding to live hippocampal neurons, the patient's serum inhibited proliferation of neuroblastoma cells, suggesting the serum contained immunoglobulin or other immune or drug factors that had direct effects on neuronal-like cells.

There are a number of approaches that could have improved our understanding of this notable case, such as longitudinal CSF testing, the use of purified immunoglobulin rather than serum testing in the pathogenic experiment, and pathologic and molecular examination of the tumor. Regardless, this thought-provoking case sheds light on the initiation and persistence of CNS autoimmunity.

STUDY FUNDING

No targeted funding reported.

DISCLOSURE

Russell Dale has received speaker honoraria from Biogen Idec, Bristol-Myers Squibb, is an editorial advisory board member for *MSARD*, an editorial board member for *Neurology® Neuroimmunology & Neuroinflammation* and *European Journal of Paediatric Neurology*, received publishing royalties from Biogen and Bristol-Myers Squibb, research support from NHMRC, Multiple Sclerosis Research Australia. Ming Lim receives research grants from Action Medical Research, DES society, GOSH charity, National Institute of Health Research (NIHR UK), MS Society, SPARKS charity; receives research support grants from the London Clinical Research Network and Evelina Appeal; has received consultation fees from CSL Behring; received travel grants from Merck Serono; and awarded educational grants to organize meetings by Novartis, Biogen Idec, Merck Serono, and Bayer. Go to Neurology.org for full disclosures.

REFERENCES

1. Maris JM. Recent advances in neuroblastoma. N Engl J Med 2010;362:2202–2211.
2. Hero B, Schleiermacher G. Update on pediatric opsoclonus myoclonus syndrome. Neuropediatrics 2013;44:324–329.
3. Gambini C, Conte M, Bernini G, et al. Neuroblastic tumors associated with opsoclonus-myoclonus syndrome: histological, immunohistochemical and molecular features of 15 Italian cases. Virchows Arch 2003;442:555–562.
4. Amini A, Lang B, Heaney D, Irani SR. Multiple sequential antibody-associated syndromes with a recurrent mutated neuroblastoma. Neurology 2016;87:634–636.
5. Jarius S, Paul F, Franciotta D, et al. Neuromyelitis optica spectrum disorders in patients with myasthenia gravis: ten new aquaporin-4 antibody positive cases and a review of the literature. Mult Scler 2012;18:1135–1143.
6. Titulaer MJ, Hoftberger R, Iizuka T, et al. Overlapping demyelinating syndromes and anti-N-methyl-D-aspartate receptor encephalitis. Ann Neurol 2014;75:411–428.
7. Panzer JA, Anand R, Dalmau J, Lynch DR. Antibodies to dendritic neuronal surface antigens in opsoclonus myoclonus ataxia syndrome. J Neuroimmunol 2015;286:86–92.
8. Ratner N, Brodeur GM, Dale RC, Schor NF. The "neuro" of neuroblastoma: neuroblastoma as a neurodevelopmental disorder. Ann Neurol Epub 2016 Apr 4.
9. Thomas S, Straathof K, Himoudi N, Anderson J, Pule M. An optimized GD2-targeting retroviral cassette for more potent and safer cellular therapy of neuroblastoma and other cancers. PLoS One 2016;11:e0152196.
10. Armangue T, Moris G, Cantarin-Extremera V, et al. Autoimmune post-herpes simplex encephalitis of adults and teenagers. Neurology 2015;85:1736–1743.
11. Honnorat J, Didelot A, Karantoni E, et al. Autoimmune limbic encephalopathy and anti-Hu antibodies in children without cancer. Neurology 2013;80:2226–2232.

© 2016 American Academy of Neurology. Unauthorized reproduction of this article is prohibited.
